# Informatics applied to cytology

**DOI:** 10.4103/1742-6413.44773

**Published:** 2008-12-29

**Authors:** Liron Pantanowitz, Maryanne Hornish, Robert A. Goulart

**Affiliations:** Department of Pathology, Division of Cytopathology, Baystate Medical Center, Tufts School of Medicine, MA, USA

**Keywords:** Computer, cytology, database, informatics internet, information technology, information system, laboratory, quality, spreadsheet

## Abstract

Automation and emerging information technologies are being adopted by cytology laboratories to augment Pap test screening and improve diagnostic accuracy. As a result, informatics, the application of computers and information systems to information management, has become essential for the successful operation of the cytopathology laboratory. This review describes how laboratory information management systems can be used to achieve an automated and seamless workflow process. The utilization of software, electronic databases and spreadsheets to perform necessary quality control measures are discussed, as well as a Lean production system and Six Sigma approach, to reduce errors in the cytopathology laboratory.

## INTRODUCTION

The Papanicolaou test (Pap test) is a highly successful, widely used, and cost effective method for the early detection of cervical dysplasia and cancer. However, Pap tests are not infallible, and emerging technologies have been developed to improve their diagnostic accuracy. The shortage of skilled cytotechnologists to screen and diagnose Pap slides has provided an additional impetus to develop automated laboratory instruments and screening systems.[1] Laboratory informatics, the application of computers and information systems to information management in the pathology laboratory, is an essential component of the cytology laboratory. These systems play a key role in the preanalytic (e.g. accessioning of a specimen), analytic (e.g. performance of special studies) and postanalytical (e.g. issuing an addendum report) test processes.[2] Providing information in a manner that is most effective for patient care is the primary mission of the cytology laboratory. In addition, data is used for the documentation of quality control (QC) measures and quality assurance (QA), performance improvement, outcomes studies, and research.[3–5]

Following the 1999 report on medical errors, from the Institute of Medicine, measures to improve patient safety and reduce errors have been applied to several medical fields, including Pathology.[6] Informatics and the use of information technology has assisted in addressing many patient safety goals and error reduction efforts by improving consistency and standardization within the laboratory, minimizing human intervention (e.g. automation), improving specimen and slide identification (e.g. barcoding), as well as facilitating peer review of cases.[4] Mislabeling errors introduced by manually labeling items (e.g. requisitions, specimens, slides) can be avoided by implementing barcoding or radiofrequncy identification in the laboratory. Barcoding also provides a tracking mechanism in the laboratory, with regard to both productivity and the exact location of orders/specimens/slides in the workflow process.

Despite the overwhelming interest in the development of several computer based technologies in the last several years, the role of automation in cytology has remained controversial.[7] Much of this stems from laboratories not knowing how to incorporate automation in the routine practice of cytology. Around a decade ago, only 12% of cytology laboratories surveyed in the United States were engaged in automated cytology. They predominantly used it for QC measures.[7] Cytology was arguably one of the first fields of pathology to embrace automation. Today, with reduced costs and increased education, there is wider acceptance of these technologies in Australia and in many developing countries, particularly with regard to computer assisted cervical screening.[5,8–9]

To the best of our knowledge, there has been no review, to date, on the topic of informatics as it applies to the field of cytopathology. Therefore, the aim of this review is to demonstrate how the modern cytology laboratory has capitalized on computers and information systems to ensure quality enhancement and improved productivity in the daily tasks of processing, screening, interpreting, and reporting Pap tests, thereby improving patient care. A Lean production system approach is discussed, showing how this approach can further help reduce errors in the cytology laboratory.

### Laboratory information systems

The laboratory information system (LIS) is the core of many cytology laboratory operations. Its functions include workflow management, specimen tracking, data entry and reporting, assistance with regulatory compliance, code capture, interfacing with other systems, archiving, inventory control, security, and providing billing information.[2,10–11] The LIS also functions as a large database and supports workflow in all steps (preanalytic, analytic, postanalytic) of the laboratory testing process. The components of the LIS include hardware (e.g. servers), peripherals (e.g. instruments, printers), a network, interfaces (e.g. links to other information systems), database(s), and software (e.g. database management system). If used properly, the LIS can be leveraged to improve efficiency, enhance productivity, reduce staff needs, and facilitate automation.

### Quality control and assurance

It is well known that maintenance of QC measures help to detect, reduce, and correct deficiencies in Pap test analysis.[12–17] QC refers to a set of procedures intended to ensure that the preparation, interpretation, and reporting of cytology specimens meets specified quality criteria. It is typically combined with quality procedures focused on the precompletion tasks. It is typically combined with quality procedures focused on the pre-completion tasks, prior to final reporting of the cytologic interpretation or diagnosis. Quality assurance is a retrospective tool that measures the success of processes. All cytology laboratories should have prior to the final reporting of the cytologic interpretation or diagnosis. All cytology laboratories should have in place a comprehensive QC/QA program designed to monitor and evaluate the overall quality of their testing process.

In the United States, the federal government defined a series of required QC/QA procedures when the Congress passed the Clinical Laboratory Improvement Amendments (CLIA) in 1988. These CLIA standards formed the minimum foundation of modern cytology QC/QA in the USA. Along with other individualized laboratory measures, they intended to ensure the accuracy, reliability and timeliness of patient test results. This process needs to be ongoing, continually evaluating factors that lead to accurate and reliable testing. With the availability of external QC measures,[18] cytology laboratories in developing countries can also benefit from participating in these procedures.[19] Utilization of spreadsheets and databases to perform QC measures is the key to the successful operation of many cytology laboratories. Moreover, in order to meet certain regulatory requirements and maintain laboratory accreditation, databases within the LIS are frequently relied upon and manipulated to perform quality measures.

The introduction of electronic record keeping has added a novel dimension to the practice of QA in contemporary cytopathology. It has ushered in a new paradigm of information management. Electronic data extraction and manipulation can be both convenient and complicated, often demanding computer-savvy trained end users, who must be willing and able to continually adapt in an environment of rapid change of both software and hardware solutions. Electronic monitoring of quality indicators has provided us with an increased range of opportunities to expose the nuances of the science and art of cytopathology. Quality Assurance methodologies are designed to continually improve the diagnostic accuracy of both the cytotechnologist and the cytopathologist, with anticipated reduction, but not elimination, of a baseline false negative rate.

### Electronic databases

Compared to paper records, electronic data has tremendous advantages, including ease and efficiency of qualitative and quantitative data analysis, standardizing and structuring the reporting of results, rapid transmission of information, efficient integration and consolidation of multiple health records, and timely financial transactions (i.e. billing). Electronic data storage also requires far less physical storage space. Moreover, multiple users may remotely access electronically stored information.

With regard to disaster recovery of data, electronic backups allow for relatively quick re-institution of data. The limitations of electronic data include obsolescence of hardware and software technologies, discontinuation of support services, the potential for easy intrusion on patient privacy, lack of end user education, and the ‘garbage in, garbage out’ (GIGO) principle. Several of these limitations can be mitigated through training and adherence to specific patient privacy guidelines (e.g. the Health Insurance Portability and Accountability Act (HIPAA) of 1996 in the United States).

Storing and retrieving data most commonly involves the utilization of a relational database. Relational databases consist of multiple tables of data, each organized into columns (fields) and rows (records). Each table must have a unique indicator or primary key. Primary keys are a column or a set of columns that uniquely identify all the rows in a table. Each row has a different primary key value and null values are not allowed. Data is linked through commonly shared fields. Linking data can be achieved by means of queries, which are software commands that search databases for particular pieces of information. A valuable application of queries lies in extracting data across multiple patients' records. For most laboratory software solutions, if new tables or entries on existing tables are added, future reports will pull data only from the date that the change was implemented forward. For this reason, the initial set up of database tables is crucial and time invested in optimizing the database design is time well spent.

### Spreadsheets

Basic spreadsheets permit users great flexibility in terms of data manipulation. Spreadsheets offer a dynamic quality to the data, as data can be charted, sorted, and organized into tables. Most laboratory software solutions accommodate the downloading of data into spreadsheets. Frequently, the cytology laboratory is dependent upon multiple disparate software systems, necessitating the need for interfaces. However, an inexpensive spreadsheet application (e.g. Microsoft Excel) may often suffice, particularly if data can be downloaded into a common platform like text (txt) or comma separated value (csv) files. The process of matching data relies on the principles of a relational database, requiring some form of unique indicator for each data set being matched. Once the desired spreadsheet is complete, contingency tables, which offer opportunities for further statistical analyses and graphs, can be generated.

### Quality indicators

Common QC/QA indicators used in the cytology laboratory are amenable to assessment by electronic methods. Such indicators may include, but are not limited to, test management and reporting systems, electronic data integrity, workflow, cytopreparation, screening and performance, postanalytical monitors (including continuing education and remedial training), and client satisfaction. Electronic tracking of QC/QA indicators can be done either within the LIS and/or by exporting data from the LIS (e.g. using common spreadsheet software).

#### Test management and reporting systems

This process includes monitoring several variables as a part of a QC/QA program. Laboratory requisition completeness can be assessed upon arrival of the specimen in the laboratory and the problems documented by the accessioner. Most software solutions have fields where QA comments can be entered. Periodic reports can be run to list occurrences and identify trends with any particular physician office sending specimens to the laboratory. Identification of problems could prompt consideration for clinician education and/or redesign of requisition forms. The timeliness of specimen transportation can be assessed by comparing the date of specimen procurement with the date received by the laboratory. Specimen rejection incidents and labeling errors should be documented, and regular reports run to monitor specimen rejection frequency by the clinician's office. Comments entered within available QA fields could be included in the final report sent to a clinician. Electronic monitoring of lost specimens is best accomplished with a simple spreadsheet log, since such specimens would not be accessioned into the LIS database.

#### Electronic data integrity

Electronic data integrity is a quality indicator that includes common network security and features to ensure that data is entered in a standardized format. Common security measures may include regular back up of data, password protection, data encryption, as well as the use of antiviral software, firewalls, and audit trails. These measures help meet the security and privacy needs related to health data, as proposed in the HIPAA.[20] Dictionaries (maintenance tables) can be built to determine which LIS users have privileges to specific fields. Assigning users different privileges in the LIS allows only certain individuals (e.g. a pathologist) to finalize and sign out abnormal gynecologic diagnoses. Audit trails can be used as a part of a comprehensive QC/QA program to ensure that users only access information relevant to their job duties. Integrity of finalized reports typically entails an electronic securing function, so that content cannot be changed or added to, except in the form of an addendum to an existing report or an amended report, thereby ensuring preservation of the original report.

Data entered using standardized language for each field is more efficiently retrieved and easily analyzed. For this reason, the use of standardized language is encouraged in the practice of cytopathology. Standardized data entry for diagnostic interpretations can be assisted through the use of coded comments, which ensure uniformity of the reporting language. In settings where standardized diagnostic terminology exists and is widely accepted, such as The Bethesda System for the reporting of Pap test results,[21] coded comments allow for quick data entry and unambiguous result reporting to the clinician. Although an internationally accepted unified terminology for the reporting of Pap tests is still required, many developed countries have adopted The Bethesda System or a slight modification thereof.[22–24] In other settings, such as fine needle aspiration biopsies and nongynecologic specimens, greater flexibility in the use of language is frequently needed. Reports that cue from standard codes provide an efficient means to extract data, enabling swift calculations of abnormal rates and breakdown of diagnostic categories for comparison against benchmark data. This can be done for the entire laboratory, as well as for individual cytotechnologists and cytopathologists.

Mandatory entries are useful features present in the LIS. With regard to Pap test reporting, for example, mandatory entries may include the following: specimen adequacy statement, primary diagnosis, billing codes, name of the cytotechnologist (primary screener) and initial diagnosis, and, if the case is forwarded for pathologist review, the pathologist's name and final diagnosis. With such a system in place, reports cannot be issued unless all the mandatory fields are completed. Ideally, the primary cytology diagnostic code should always remain assigned to the case, regardless of what is typed in lieu of, or in addition to, the primary coded remark. This process will ensure that future data analyses based upon retrieval by diagnostic codes are accurate and meaningful. Queries based upon the text entered (i.e. natural language search) are possible with many current laboratory information systems. However, something as simple as typographical errors may interfere with searches based on text alone. This can be avoided by utilizing common built-in software features such as a spell check and automated comments in the LIS.

#### Workflow indicators

Turnaround time (i.e. the time it takes to provide the patient's caregiver with a cytology report from the time the Pap test specimen was received within the laboratory) provides an excellent gauge of workflow management.[25] As quality in reporting and diagnostic accuracy are very often assumed by both the clinician and the patient, turnaround time unfortunately serves as the major ‘quality’ indicator to both clinical office personnel and the public. As such, the impact of efficiently managing the laboratory to effectively reduce this time cannot be overstated. Reports can be generated to calculate turnaround time, identify outliers, investigate the cause of a delay, and implement corrective actions. Trail sheets can be used to track locations of specimens, should they be sent to different departments or locations outside the laboratory (e.g. to a reference laboratory).

Retrospective reviews may involve randomly selecting a predetermined number of cases from the previous month and checking them for timely retrieval, appropriate storage, and reporting accuracy. Errors should be documented and procedures reevaluated. If necessary, they should be revised. In the United States, CLIA regulations mandate a retrospective review of all negative Pap tests for the last five years for which current cases are diagnosed with a high grade squamous intraepithelial lesion (HSIL) or above (i.e. cancer). A five-year retrospective review has been noted to highlight problem areas for laboratory education and quality improvement efforts, although liability concerns have been reported to be a detrimental consequence of this regulation.[26] Clarification that amended reports be issued only when current patient care is affected, as has been proposed by some authors, should aid laboratories in complying with this regulation.[26]

Clinical Laboratory Improvement Amendments (CLIA) also requires the cytology laboratory to provide and document whether alert information (typically in the form of a medical director letter) was sent to providers, with regard to all patients with a Pap test diagnosis of HSIL (or carcinoma) in which there is no record of further patient follow-up in the pathology or hospital information system. This ensures that those patients at high risk for cervical cancer get appropriate timely intervention and treatment. In order to meet this requirement, laboratory software can be designed to search for cases of HSIL (or carcinoma) during a specific time period (e.g. prior three or four months) and then identify any cytology or surgical specimen(s) that have occurred since that designated period. In the United States, although a Pap test interpretation of HSIL is the trigger for this federal requirement, many laboratories include other ‘lesser’ Pap test interpretations as triggering events, specifically those which also carry a significant risk of the patient harboring a high-grade precancerous cervical or vaginal lesion, or potentially invasive carcinoma. In this way, the quality monitor can be modified to meet the goals and expectations of one's local medical community (and medico-legal environment), based upon the judgment of the laboratory medical director. If no such subsequent specimens can be found in the LIS database, letters that alert the appropriate clinician to this situation and request follow-up information can be generated.

#### Screening and performance indicators

Quality indicators that are related to screening and performance are both subjective and objective in nature. An important component of any accepted QC/QA program in cytology includes provisions for setting up the maximum workload for individual cytotechnologists. This can be determined by evaluating diagnostic accuracy as it compares to productivity, and is typically described in terms of slides per hour. Federal law in the United States requires that cytotechnologists manually document the number of slides screened within each 24-hour period, and the number of hours spent screening each day. Guidelines prohibit screening more than 100 slides (conventional Pap smears and/or liquid-based Pap tests) over an 8-hour period, and not more than 12.5 slides per hour. It is generally accepted that higher per hour and per day slide counts are permissible when screening Pap tests which have been imaged and screened by computer systems, as they allow the cytotechnologist to review a smaller surface area microscopically per negative slide. However, this matter is complicated (e.g. there is a learning curve inherent with new technology that needs to be taken into consideration) and has been addressed in the guidelines published by the Cytopathology Education and Technology Consortium (CTEC).[27] In a similar fashion, nongynecologic liquid-based preparation slides also count as half-slides in such daily counts. Slides per hour can be calculated by adding the total number of slides and dividing them by the total hours spent screening, which is determined by subtracting time spent doing nonscreening activities from the hours worked. Software solutions can be used to ensure that workload limits are not exceeded. For example, once the daily workload limit has been reached for an individual cytotechnologist, the LIS will not allow him to sign out additional cases.

Common indicators of diagnostic accuracy include rescreening measures. Rescreening is mandated by the CLIA in the United States, but has also been implemented as a measure of QA in screening Pap tests in other countries.[28] This includes a 10% random rescreen of negative Pap tests and a rescreening of a specified percentage of negative cases defined as ‘high risk’. All rescreening should be performed by a supervisory level cytotechnologist or a pathologist. Several studies have shown that a 100% rapid rescreening method is more efficient at detecting false negative results than 10% random rescreening or rescreening on the basis of clinical criteria, and this has been recommended as an internal QC method.[29–30] However, this may not be practical in a large cytology laboratory screening a high volume of cases or one with limited staff. Software can be relied upon to allow the percentage of cases pulled for QC review to be adjusted and this can be set differently for individual screeners. Situations where this may be useful would be performance indicators that mandate closer inspection of work quality, a tool for remedial training, or for new hirees whose work quality is not yet fully ascertained. Quality Control (QC) rescreening is most useful for exposing screening errors, as opposed to diagnostic errors. Accordingly, software often has data entries that allow the degree of error to be defined (e.g. minor disagreement for the missed presence of Candida or major disagreement for a missed squamous intraepithelial lesion/SIL).

A prospective random 10% rescreen (re-examination prior to reporting), typically determined by laboratory software, automatically marks for QC each 10th consecutive negative case entered by a primary screening cytotechnologist on a lab-wide basis. If the laboratory has many cytotechnologists signing out cases simultaneously, the 10% should represent a random selection that is unpredictable from the perspective of each cytotechnologist. Once selected for QC, the laboratory software should ensure that the case cannot be modified by the primary screening cytotechnologist. This case must also have a documented review by a person at an appropriate level, defined in the person dictionary (e.g. supervisory cytotechnologist or pathologist). Reports should be generated to verify that each cytotechnologist has indeed met the minimum requirement of 10% QC rescreen of negative Pap test cases.

Cases are designated as high risk, based upon past and/or current history of abnormal signs, symptoms and/or pathologic findings. The definition of ‘high risk’ varies widely between laboratories, as it is subject to continual adjustment, based upon the specific patient population served, evolving scientific data, development of new clinical condition states (e.g. HIV/AIDS), new testing modalities (e.g. HPV DNA testing), and the judgment of the laboratory medical director. Software solutions can automatically designate cases as high risk, based on database searches for particular entries within certain fields with consideration for a specific time period (e.g. ‘History of LSIL’ in current field of ‘clinical history’, or a previous specimen coded as abnormal in the primary diagnosis interpretation field within the past three years). Computer-assisted designation of high risk cases greatly increases the likelihood that high risk cases are correctly identified to be a part of a secondary review process prior to sign out. Since cases defined as ‘high risk’ are theoretically at higher risk for containing abnormalities, patient care is improved with the (typically small) proportion of negative cases by primary review that are found upon secondary review to contain abnormal findings.

A large component of workload assessment for cytotechnologists involves comparing diagnostic agreement between the cytotechnologist and the reviewing pathologist for each case forwarded for pathologist review. This is most critical for the negative Pap test by cytotechnologist review. As cytotechnologists function not only as Pap test screeners but also as Pap test diagnosticians, solely evaluating and reporting the majority of negative Pap tests and thus the overall majority of all patient Pap test results, the evaluation of their ability to accurately determine a Pap slide as negative is crucial. All Pap tests initially deemed negative for intraepithelial lesion or malignancy by cytotechnologist review, which then undergo second review due to standard QC practices (10% or high risk rescreen) or pathologist review due to the presence of reactive changes, serve as QA data which must be analyzed. Pivot tables, created after downloading data into spreadsheets, are useful tools to cross reference diagnoses by cytotechnologists and pathologists and for analyzing diagnostic correlations and discrepancies of Pap tests, or in addition nongynecological cases. Areas of cytotechnologist over-interpretation and more importantly under-interpretation (potential false negative cases) can be easily assessed on both an individual cytotechnologist or laboratory-wide. Software systems can also allow for individual cases to be easily retrieved for continuing education purposes.

Additional performance monitors that can be tracked include the rates for frequencies of diagnostic categories (e.g. atypical squamous cells of undetermined significance or ASC-US) and the ASC/SIL rates,[31] which can be tabulated on a monthly, quarterly, and/or six month basis for each cytologist and compared against the department as a whole, as well as benchmark data where available. Remedial action may be taken if an individual average exceeds a predetermined variance from the norm (e.g. two standard deviations from the laboratory average). If rates fall outside this range, this will prompt a review and appropriate continuing education of individual and/or lab performance by the medical director. With the increasing availability of Human Papilloma Virus (HPV) DNA testing of liquid-based Pap test material, the ASC-US HPV DNA positivity rate can similarly serve as a useful quality indicator.[32] This rate is particularly informative, with regard to pathologist performance and appropriate utilization of the ASC-US category, providing excellent and timely feedback with well-accepted target performance benchmarks. Performance beyond two standard deviations (SDs) of the mean, for HPV-positive rates in ASC-US Pap tests, has been recommended by some authors to prompt reassessment of diagnostic criteria used in the evaluation of Pap tests and/or investigation of the prevalence of HPV positivity in the population from which the Pap tests are obtained.[33] One can also note the proportion of particular diagnostic categories forwarded for pathologist review by each cytotechnologist, and observe, with discrepancy logs, the percentage of their negative cases signed out by the pathologist as abnormal.[34]

For QC purposes, cytology laboratories need to compare Pap test and cervical biopsy reports (if available) and determine the cause of any discrepancies.[35–36] Such cytologic-histologic correlation should be compiled at regular (e.g. six month) intervals. Continuous monitoring of cytologic-histologic correlation data has been shown to be associated with improvement in cervical cancer screening performance (e.g. higher predictive value of a positive Pap test and superior sensitivity).[37] This entails comparing Pap test data to database searches of subsequent surgical specimens (e.g. colposcopic biopsies or excisional cone procedures). The tissue specimen is often regarded as the ‘gold standard’, although colposcopic examination, even with the most experienced colposcopist, has relatively limited sensitivity. With the previously described QC procedures, specifically 10% and high risk rescreen and cytotechnologist vs. pathologist diagnosis correlation data, coupled with Pap test vs. subsequent biopsy correlation data, potential false negative and false positive Pap tests can be identified and assessed for both individuals and the laboratory as a whole. The subgroup of potential false negative cases should be examined for assessing issues of sampling (cells representative of the lesion are absent or in very low number), as well as examination of the potential false positive Pap slides for confirmation of the abnormal finding as originally diagnosed (colposcopic biopsies missed the lesion). In the latter situation, if the abnormal Pap finding is confirmed, it is imperative to reinforce this to the clinician, so that further biopsy, LEEP excision, or close Pap follow-up can be scheduled. If Pap case selection by the cytotechnologist is random, as well as an equally distributed or random interaction of the cytotechnologist and pathologist pools, then the rates of false negative Pap interpretation can be compared and contrasted between individual cytotechnologists. In large cytology laboratories, interpreting thousands, if not hundreds of thousands of Pap tests, this can only be accomplished utilizing the LIS through QC/QA software tools. One common indicator, amongst others, is the false negative rate (FNR) (also known as the false negative fraction or false negative proportion), which is the proportion of abnormal Pap tests that are falsely negative, i.e. FNR = false negatives/(true positives + false negatives).[13]

### Lean and Six Sigma approach

The aim of most laboratory operations is to deliver services at low cost, high quality, and in a safe manner. However, no matter how well we standardize our procedures or how hard we try to perfect the testing process, errors are bound to occur. It is well known that eliminating medical errors is a matter of improving systems, not just people.[4] While statistical QC works well, as shown above, it is only helpful for those tasks in which numbers are generated. Lean manufacturing and Six Sigma are both methodologies used in continuous process improvement and ultimately total quality management. Lean principles, that revolutionized manufacturing, are bringing dramatic changes to the healthcare sector.[38–41] Based on the Toyota Production System, Lean manufacturing is a systematic approach to identifying and eliminating waste. Waste may be related to one of several factors such as transportation, inventory, waiting, overproduction, and overprocessing. Six Sigma is a business-driven, multifaceted approach to quality improvement, based on error reduction, improvement in testing processes (including reduction in process variation), and financial targets (i.e. cost reduction or profits).

These business strategies use quality management tools such as value stream mapping, so-called 5S, Kaizen, and DMAIC. Value stream mapping entails diagramming the detailed activities of the process, showing the flow of specimens and information through the laboratory. Each step should be evaluated for its value contribution to the final end product and its effect on quality. If the step is a non-value-added step it should be considered waste, and, therefore, eliminated. Laboratory staff having to accession and/or retrieve data in more than one information system may be considered an example of overprocessing. Notably, certain non-value-added steps may be necessary, solely for licensure or accreditation purposes. The 5S principle stands for five S's, viz. sort (removal of all unnecessary items from the work environment), simplify (label and arrange key items in the area), shine (clean the area), standardize (develop a consistent way of performing the tasks), and sustain (develop checklists and audits to maintain the improvement). Kaizen (Japanese for ‘improvement’) refers to the continuous incremental improvement of Lean activity to eliminate waste. DMAIC stands for define (process improvement goals), measure (the current process and collection of relevant data), analyze (data to verify cause-and-effect relationships), improve (optimize the process based upon data analysis), and control (correct deviations from target before they result in defects). Clearly, a large part of the appeal of a Six Sigma approach in the pathology laboratory is its reliance on accurate data and measurement. Although Six Sigma software packages are available, the LIS can be leveraged to help satisfy several of these requirements.

Once the aforementioned methodologies are applied to the cytology laboratory, the process must be monitored to determine whether the solution was successful. For example, success can be measured by improved turnaround time or reduced operating costs. After introducing the Toyota production system methods at one medical center, researchers reported improved Pap test quality (5.2% reduction in Pap tests lacking a transformation zone, 3.9% decrease in ASCUS diagnosis, and 1.68% decrease in error per correlating cytologic-histologic specimens), after eight months.[42] The same group of investigators subsequently showed that disseminating Lean methods in five clinician practices resulted in improved Pap test quality and diagnostic accuracy.[43]

## CONCLUSIONS

Laboratory informatics is critical to meet current and future challenges. These include growing workloads, the shortage of laboratory technologists, patient safety (i.e. error reduction), cost containment, subspecialty centralization, increased demand for molecular testing, and personalized medicine. Many of these challenges can be met by leveraging existing and advancing technologies, such as improving the integration of disparate information systems, automation, specimen tracking, electronic document management systems, and streamlining procedures. The increased use of liquid-based methods for cytopathology has provided the platform for several new advances, including automation in the cytology laboratory. Adding Lean principles to existing quality improvement programs optimize specimen and information flow, increase productivity, and reduce chronic waste. This should translate into improved operating performance (higher productivity and lower cost), better turnaround time, more efficient space utilization, and reduction of errors.

Finally, we believe that training programs for cytotechnologists and cytopathologists need to begin providing formal informatics training and instruction with regard to these new technologies.

## COMPETING INTERESTS

The authors declare that they have no competing interests.

## AUTHORS' CONTRIBUTIONS

All authors contributed equally to all aspects of this manuscript.
